# Accessibility of health care experienced by persons with dementia from ethnic minority groups and formal and informal caregivers: A scoping review of European literature

**DOI:** 10.1177/14713012211055307

**Published:** 2021-12-08

**Authors:** Gözde Duran-Kiraç, Özgül Uysal-Bozkir, Ronald Uittenbroek, Hein van Hout, Marjolein I. Broese van Groenou

**Affiliations:** Health and Social Care Department, 8771 Windesheim University of Applied Sciences, Zwolle, Netherlands; Vrije Universiteit, Amsterdam, Netherlands; 6984 Erasmus School of Social and Behavioural Sciences, Department of Psychology, Education and Child Studies, Erasmus University Rotterdam, Netherlands; Health and Social Care Department, 8771 Windesheim University of Applied Sciences, Zwolle, Netherlands; Departments of General Practice & Medicine of Older People, 522567 Amsterdam University Medical Centers, Amsterdam, Netherlands; Vrije Universiteit, Amsterdam, Netherlands; Department of Sociology, Faculty of Social Sciences, 1190 Vrije Universiteit, Amsterdam, Netherlands

**Keywords:** dementia care, migrants, health services accessibility, nurses

## Abstract

The number of persons with dementia from ethnic minority backgrounds is increasing. However, ethnic minority groups use health care services less frequently compared to the general population. We conducted a scoping review and used the theoretical framework developed by Levesque to provide an overview of the literature concerning access to health care for ethnic minority people with dementia and (in)formal caregivers. Studies mentioned barriers in (1) the ability to perceive a need for care in terms of health literacy, health beliefs and trust, and expectations; (2) the ability to seek care because of personal and social values and the lack of knowledge regarding health care options; and (3) lack of person-centered care as barrier to continue with professional health care. Studies also mentioned barriers experienced by professionals in (1) communication with ethnic minorities and knowledge about available resources for professionals; (2) cultural and social factors influencing the professionals’ attitudes towards ethnic minorities; and (3) the appropriateness of care and lacking competencies to work with people with dementia from ethnic minority groups and informal caregivers. By addressing health literacy including knowledge about the causes of dementia, people with dementia from ethnic minorities and their informal caregivers may improve their abilities to access health care. Health care professionals need to strengthen their competencies in order to facilitate access to health care for this group.

## Introduction

People who migrated to Europe for economic reasons in the 1960s are now reaching the age at which a substantial group develops dementia (APPGD All-Party Parliamentary Group on Dementia, 2013; Alzheimer Europe, 2018). According to Canivelli et al. (2019), nearly 6.5% of all cases of dementia in Europe are expected to involve foreign-born persons. A proportion of these people are so called ethnic minorities (EM) with different cultural background and reduced access to care (Canevelli et al., 2019). For this study, we define EM people as European migrants, non-western migrants, and non-migrant minorities living in Europe. Some studies show even a higher increase for the number of persons with dementia from EM groups compared to the general population (Alzheimer Nederland, 2014; APPGD [All-Party Parliamentary Group on Dementia], 2013; Nielsen et al., 2015). One of the main reasons for this difference is the vulnerability of EM groups to dementia due to accumulating risk factors, for example, diabetes ,cardiovascular diseases, and low literacy, which have been indicated as causes for the higher dementia prevalence within the EM population (Bindraban et al., 2008; Parlevliet et al., 2016; Rosenbaum et al., 2008; Uitewaal et al., 2004).

EM groups in Europe differ from the non-migrant population in their utilization of care and support. Primary health care is in some countries like Denmark, Germany, Norway, and the Netherlands more frequently used by persons with EM backgrounds compared with the native population (CBS, 2018; Diaz et al., 2014; Denktas et al., 2009; Glaesmer et al., 2011; Nielsen et al., 2012). On the other hand, people from EM backgrounds utilize other forms of health care services less frequently (Denktaş et al., 2009). Research shows that EM groups experience more stigma for common mental disorders (Eylem et al., 2020). Moreover, despite well-developed health care systems in some European countries, such as the Netherland and Denmark, both countries report low utilization of nursing homes by EM groups (Alzheimer Nederland, 2014; Stevnsborg et al., 2016). Furthermore, only 1% of Moroccan and 7% of the Turkish older adults with dementia use home care, as opposed to 16% of the non-migrant Dutch adults (Alzheimer Nederland, 2014).

The underuse of dementia care by EM groups might be a result of a mismatch between supply and demand side of access to health care. The development of intercultural care may be hindered by this mismatch. Intercultural care means responding to and respecting the cultural identities of people and understanding what is generally important to them, without losing sight of individuals amongst generalizations and stereotyping (Alzheimer Europe, 2018). Ethnocentrism (judging other cultures by the standards of one’s own culture), lack of culturally appropriate services, cultural beliefs surrounding dementia and care, limited knowledge about dementia, stigma and shame, and negative evaluations of mainstream services are a few explanations given for differences in access to and utilization of dementia care (Alzheimer Europe, 2018). Another explanation for underuse of health care by EM groups is the assumption that informal caregivers with EM backgrounds are culturally obliged to provide care and they are able to do so due to support from large extended families. This may lead to informal caregivers not being offered support and services they require (Parveen et al., 2017). Informal care is defined as unpaid care provided to the person with dementia by a person within the patients’ social network, where formal care refers to paid health care services provided by a health care organization or care professional (Triantafillou et al., 2010). Although several other studies reported barriers regarding access to and utilization of dementia care, a broad overview is lacking. Therefore, in our scoping review, we aim to provide an overview of potential barriers and facilitators present in the process of EM groups utilizing dementia care, and to answer the following research question: “What are the perceived abilities and inabilities of people with dementia from ethnic minority backgrounds and their informal caregivers to access care, and how do health care professionals and specifically nurses experience offering dementia services to ethnic minority persons and their informal caregivers?”

## Theoretical framework

**Figure 1.**
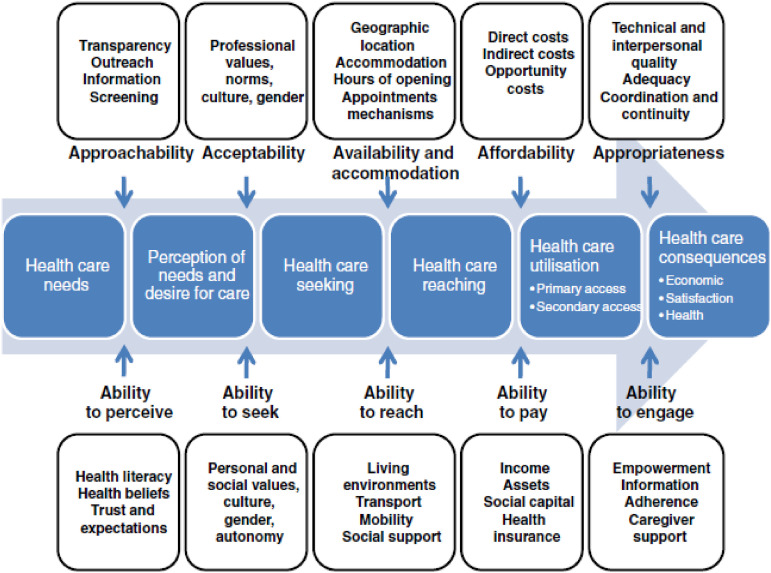
Conceptual framework of access to health care by Levesque et al. (2013).

We use the framework of Levesque et al. (2013) to identify different steps needed to access and utilize health care services (see Figure 1). This framework proved to be useful in previous research by Suurmond and colleagues (2016). Each step relates to abilities required to navigate the care landscape (the demand side), and determinants of accessibility from an organizational viewpoint (the supply side).

### Supply side of access to health care

The first of the five dimensions on the supply side is labeled “approachability” and refers to transparency, information regarding available treatments and services that can contribute to the approachability of services. “Acceptability,” the second dimension, refers to factors that determine the possibility for people to accept the service, and the perceived appropriateness for the persons to seek care. Important in this dimension are values and norms of the professional, culture, and gender of the professional, and the organizational culture. Acceptability relates to cultural and social factors determining the possibility for people to accept the aspects of the service such as the sex or social group of the provider (Levesque et al., 2013). “Availability and accommodation,” the third dimension of accessibility of services, includes determinants that define the extent to which health care services can be reached both physically and in a timely manner. Another dimension on the supply side of access to health care is the “affordability” of care services. The final dimension is “appropriateness” which relates to the fit between services and clients’ needs.

### Demand side of access to health care

The five dimensions on the supply side correspond with five dimensions on the demand side, referring to what abilities potential clients need to access health care. The approachability of health care requires the “ability to perceive” a need for health care and consists of determinants such as health literacy, health beliefs, trust, and expectations. The acceptability of health care requires the “ability to seek” health care and is made up of the client’s personal and social values, culture, gender, and autonomy. It also includes knowledge about health care options. For example, a society forbidding casual physical contact between unmarried men and women would reduce acceptability of care and acceptability to seek care for women if health service providers are mostly men. (Levesque et al., 2013)

The dimension of availability and accommodations requires the ‘ability to reach’ health care. Affordability requires the ‘ability to pay’ for health care and it describes the capacity to generate economic resources to pay for health care services. Appropriateness of health care services requires the “ability to engage” in health care. It relates to the participation and involvement of the client in decision making and treatment decisions. For this study, we chose to exclude the ability to pay, as there are too many differences between national health care systems and as such financial aspects that affect the accessibility of dementia care.

## Methods

Following the methodology for conducting a scoping review according to the Joanna Briggs Institute (2020), we used the framework of Arksey and O’Malley (2005). According to this framework, a scoping review aims to discover research gaps in the existing literature and allows the inclusion of all types of studies and provides an overview of the breadth, rather than the depth of evidence. The framework to conduct a scoping review consists of different stages: (1) identifying the research question, (2) identifying relevant studies, (3) study selection (4) charting the data, (5) collating, summarizing and reporting the results; and the additional stage (6) consultation (Arksey & O’Malley, 2005). Although a scoping review does not necessarily require an appraisal of the methodological quality of the studies, we used the Mixed Methods Appraisal Tool (MMAT) (Hong et al. 2018).

### Identifying relevant studies

Key concepts and search terms were identified in cooperation with a librarian (LS). The following search key concepts and associated synonyms were used: dementia, ethnic minorities, and nurses. Nurses were included in the search strategy, but we found relevant papers not including nurses only, but also other health care professionals. We therefore broadened the scope and also included papers that mention nurses and other health care professionals. Subsequently we determined a search strategy with synonyms for the key concepts and chose the databases PubMed, Embase, Cinahl, PsychInfo, and Scopus. The literature search was conducted in October 2019. Additionally, we screened the Alzheimer Europe report “The development of intercultural care and support for people with dementia from EM groups” (Alzheimer Europe, 2018).

### Study selection and inclusion criteria

**Figure 2.**
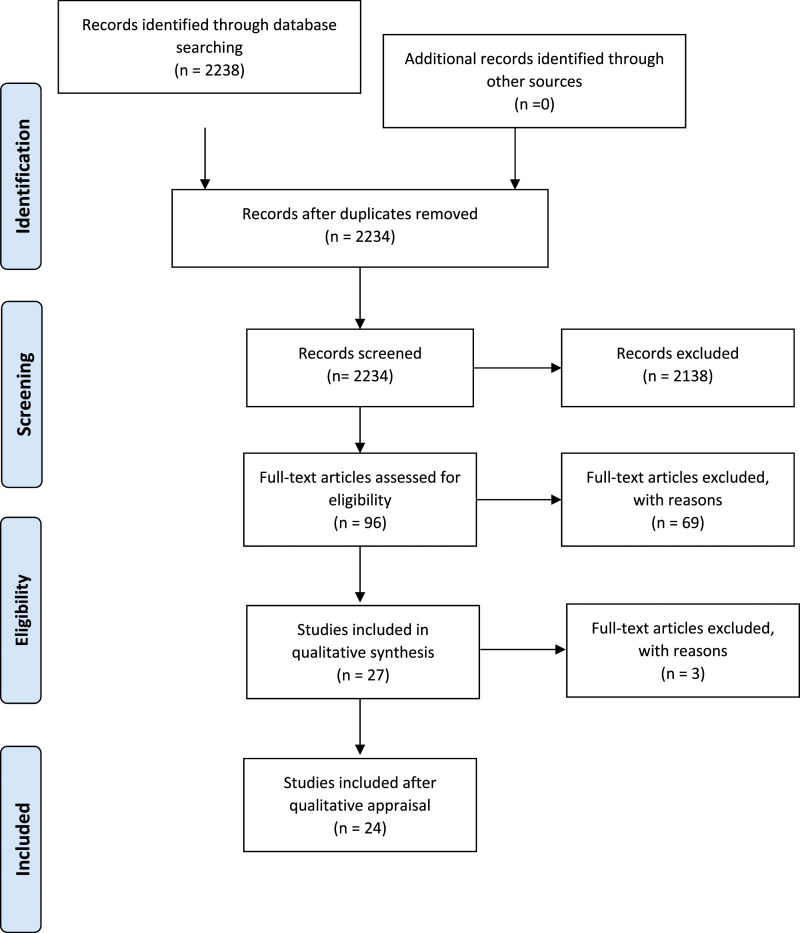
Prisma flow diagram.

The criteria for inclusion of empirical studies were: (1) reporting on dementia among persons from EM groups living at home, (2) including information about the abilities and factors that influence persons from EM backgrounds and their caregivers to perceive a need for health care, (3) including information about the abilities of EM persons with dementia and their caregivers to seek health care and the factors that influence the search for health care, (4) including information about the abilities and the factors that influence engagement in health care on the demand side, (5) published in 2000 or later to be sure that we include the latest insights and knowledge and to exclude outdated studies, (6) focus on people with dementia from EM groups and/or their caregivers living in Europe, and (7) the paper should be written in English. We have chosen to focus on Europe in this scoping review because EM groups living in Europe generally differ in cultural and religious background from the EM groups living, that is, in the United States. We excluded all papers based on diagnostic tools, papers about people with dementia from EM groups in nursing homes and systematic reviews. We also excluded editorials, columns, book reviews, and abstracts for conferences. The first study selection was done by two independent researchers (GD and ÖU) who screened titles and abstracts. If the relevance of a study was unclear, no abstract was available or the title and abstract did not provide sufficient information for evaluation, then the full paper was read. If a paper was not available through institutional holdings, we contacted the author or obtained electronic versions of the article from other universities. Both researchers independently reviewed the full texts of the studies that were included based on the first screening. A total of 142 studies (6.36%) conflicted between both researchers. These disparities regarding inclusion or exclusion of studies were resolved through discussion and reaching consensus. This first phase resulted in a set of 27 eligible papers out of 2238 records (see Figure 2).

### Methodological appraisal

To appraise the methodological quality of the included studies, we applied the Mixed Methods Appraisal Tool (MMAT) developed by Hong et al. (2018). The MMAT has two screening questions which indicate that a clear research question must be formulated and that the collected data allows the research questions to be addressed. If at least one of these two questions is answered with a “can’t tell” or a “no,” further appraisal of the study is not appropriate and should be excluded. The other five questions are based on the type of study and assessment of their methodological quality. Each author independently appraised the methodological quality of a selection of the included studies. The first and second author appraised 14 and 13, respectively. The other authors each appraised 9 or 10 of the 27 studies. These studies were randomly assigned by author 1. Results were compared and disparities regarding inclusion or exclusion of the studies were resolved through consensus. Finally, we applied the scoring system developed by Pluye et al. (2009), which allows for a score to be calculated as a percentage (Pluye et al., 2009). 24 of the 27 included studies (89%) scored between 71% and 100%, of which 12 had a 100% score. The manual does not provide a cutoff point, and the deletion of studies with low quality is left up to the authors (Hong et al., 2018). Because the quality was lower than 70% or could not be assessed with the MMAT, we decided to exclude the studies from Jolley et al. (2009), Jutlla & Moreland (2009) and Patel & Mirza (2000). We continued with the set of 24 studies.

*Charting the data*

After the study selection, we charted key items of information obtained from the included studies, such as author(s), year of publication, study location, study design, study population, aims of the study, methodology, and important results (Arksey & O’Mally, 2005).

*Collating, summarizing and reporting*

In stage five we collated, summarized, and reported the main results of the included studies. We decided what information should be recorded and compared to be able to answer the research question. We used Levesque’s et al., (2013) framework of access to health care to chart and summarize the findings of the scoping search. The literature was organized thematically according to the different determinants of access to care from the demand side and the supply side.

## Results

### Descriptive overview

We included 24 studies, most of which were based on qualitative methodology (*n* = 22). A majority of the studies were conducted in the United Kingdom (*n* = 15). Other papers were from Norway (*n* = 5), Belgium (*n* = 1), the Netherlands (*n* = 2), and Denmark (*n* = 1). The included studies contain a wide range of EM groups and professionals working in health care. The size of the samples in the studies varied from a case study (n=1) to n=230.

Based on the conceptual framework of Levesque et al. (2013) we found three main themes: 1) health care needs, 2) health care seeking, and 3) health care utilization. These themes all contained subthemes from the demand side and the supply side of to access health care. We also searched for health care reaching in all studies, but found no results. Going beyond Levesque´s framework we also found results related to the diversity within and between EM groups (see Table 1). To enhance readability, we refer to the papers throughout the result section using their numbers given in Table 1.

**Table 1.** Overview of included studies.

						Demand side			Supply side		
Author(s), year	Study location	Aim	Design & method(s)	Study population	Diversity	Ability to perceive	Ability to seek	Ability to engage	Approachability	Acceptability	Approap riateness
1. Adamson, J. (2001)	United Kingdom	To explore awareness, recognition, and understanding of dementia symptoms in families of South Asian and African/Caribbean descent in the UK.	Qualitative in-depth, semi-structured interviews	*n* = 30 (African/Caribbean & South Asian carers)	x	x		x			
2. Baghirathan et al. (2018)	United Kingdom	To generate a grounded theory about the experiences of caregivers of people with dementia from the BAME communities	Qualitative semi-structured and focus groups interviews	Interviews: *n* = 27 Focus groups: *n* = 76 (Different South Asian communities, Chinese community and African Caribbean community)	x	x	x	x			x
3. Berdai Chaouni & De Donder (2018)	Belgium	To explore how Moroccan families facing dementia experience and manage the condition	Qualitative semi-structured interviews with informal caregivers (12) and professionals (13)	*n* = 25(Informal caregivers Moroccan; professionals 6 Moroccan and 7 Belgium)		x	x	x	x	x	x
4. Blix & Hamran (2017)	Norway	To explore healthcare professionals’ discursive constructions of Sami persons with dementia and their families’ reluctance to seek and accept help from healthcare services	Qualitative focus group interviews	*n* = 18 (Sami)					x	x	x
5. Botsford et al. (2012)	United Kingdom	To examine the experiences of partners of people with dementia in two minority ethnic communities	Qualitative in-depth interviews	*n* = 13 (Greek Cypriot & African Caribbean)	x	x	x	x			
6. Bowes & Wilkinson (2003)	United Kingdom	To examine some views and experiences of dementia among older South Asian people, as well as their families and carers, and to explore central issues of service support	Qualitative Semi-structured interviews and case studies	*n* = 11 *n* = 4 case studies (South Asian)		x	x	x	x	x	x
7. Giebel et al. (2016)	United Kingdom	To investigate how South Asians with self-defined memory problems, with and without GP consultation, construe the symptoms, causes, consequences, and treatment of the condition	Quantitative	*n* = 33 (South Asian)		x		x			
8. Hossain & Khan (2019)	United Kingdom	To explore the perspectives of Bangladeshi family carers’ knowledge and day-to-day experiences of caring for someone with dementia living in England	Qualitative semi-structured face-to-face interviews	*n* = 6 (Bangladeshi)	x	x		x			
9. Jutlla, K. (2014)	United Kingdom	To explore how migration experiences and life histories impact on perceptions and experiences of caring for a family member with dementia for sikhs living in wolverhampton in UK.	Qualitative narrative interviews	*n* = 12 (Sikh)		x	x	x			
10. La Fontaine et al. (2007)	United Kingdom	To explore perceptions of aging, dementia and associated mental health issues amongst British South Asians of Punjabi Indian origin	Qualitative Focus groups	*n* = 49 (Punjabi and Indian)		x	x	x			
11. Larsen, Normann, & hamran, T (2015)	Norway	To explore how caregivers experience collaboration in rural municipalities in northern Norway	Qualitative fieldwork (observations) and semi-structured interviews	*n* = 17 (Sami)		x		x		x	x
12. Lawrence et al. (2008)	United Kingdom	To explore the caregiving attitudes, experiences and needs of family carers of people with dementia from the three largest ethnic groups in the UK.	Qualitative in-depth individual interviews	*n* = 32 (Black Caribbean, South Asian and White British)		x	x	x			
13. Lawrence et al. (2011)	United Kingdom	To examine the subjective reality of living with dementia from the perspective of people with dementia within the 3 largest ethnic groups in the UK.	Qualitative In-depth individual interviews	*n* = 30 (Black Caribbean, South Asian, and White British)	x	x	x				
14. Mackenzie (2016)	United Kingdom	To identify the support needs of family carers from Eastern European and South Asian communities and to develop and deliver support group programs combined with advocacy support for carers	Qualitative Semi-structured interviews & field notes	*n* = 21 (Pakistani, Indian, Polish, and Ukrainian)	x	x	x	x			
15. Mountford & Dening (2019)	United Kingdom	To explore an understanding of culture and ethnic background in families’ experiences of dementia and caring using a culturagram assessment framework	Qualitative case study	*n* = 1 (Italian)		x	x	x		x	x
16. Mukadam et al. (2011)	United Kingdom	To explore the link between attitudes to help-seeking for dementia and the help-seeking pathway in minority ethnic groups and indigenous population	Qualitative Semi-structured interviews	*n* = 18 (White UK carers, South Asian, Black (African or Caribbean, Asian, and Chinese)		x	x	x			
17. Naess & Moen (2014)	Norway	To explore response processes surrounding signs and symptoms of dementia and understanding how Norwegian–Pakistani families negotiate dementia in the space between their own imported culturally defined system of cure and care, and the Norwegian healthcare culture	Qualitative observations and in-depth interviews	*n* = 22 (Pakistani)		x	x	x			
18. Nielsen et al. (2019)	Denmark	To examine barriers to accessing post-diagnostic care and support in ME communities from the perspective of primary care dementia coordinators	Quantitative	*n* = 41 (Primary care dementia coordinators)					x		x
19. Parveen et al. (2018)	United Kingdom	To evaluate whether an information program for South Asian families (IPSAF) had an impact with regard to knowledge of dementia and use of services	Quantitative & qualitative knowledge quiz Focus groups and family interviews	*n* = 42 focus groups *n* = 17 family interviews *n* = 33 knowledge quiz (Indian, Pakistani, and Bangladeshi)		x	x	x			
20. Sagbakken et al. (2018a)	Norway	To explore and describe the views and experiences of family members and professional caregivers regarding the care provided to immigrants with dementia or age-related cognitive impairment	Qualitative individual in-depth interviews, dyad interviews & focus group discussions with family members (12) and professionals (18)	*n* = 30 (Afghanistan, Pakistan, China, Vietnam, Turkey, Lebanon, Sri Lanka, and Chile)		x	x	x	x		
21. Sagbakken et al. (2018b)	Norway	To explore the challenges involved in identifying, assessing and diagnosing persons with different linguistic and cultural backgrounds from the perspective of health professionals	Qualitative individual (7), dyad interviews (3) and 3 focus group discussions (consisted of 4–6 participants)	*n* = 27 (Professionals)					x		x
22. Seabroke & Milne (2004)	United Kingdom	To explore the service-related needs of Asian older people with dementia and their carers	Qualitative semi-structured interviews, workshops, and focus groups	*n* = 7 GPs *n* = 32 statutory and voluntary sector health professionals *n* = 13 service managers *n* = 7 carers *n* = 21 nursing and residential care homes *n* = 230 members of the local Asian community		x	x	x			
23. van Wezel et al. (2016)	The Netherlands	To describe the perspectives of female Turkish, Moroccan, and Surinamese Creole family carers in the Netherlands on providing family care to a close relative with dementia	Qualitative Individual interviews and focus groups	*n* = 41 individual interviews and 6 focus group interviews (*n* = 28) (Turkish, Moroccan, and Surinamese Creole)	x	x	x				
24. van Wezel et al. (2018)	The Netherlands	To give insight into the in the explanations for dementia of Turkish, Moroccan, and Surinamese–Creole family caregivers	Qualitative Individual interviews and focus groups	*n* = 41 individual interviews and 6 focus group interviews (*n* = 28) (Turkish, Moroccan, and Surinamese Creole)	x	x					

## Health care needs: ability to perceive (demand)

Nearly all papers studying the demand side of dementia care mentioned the influence of health literacy, health beliefs, trust, and expectations regarding the ability to perceive a need for dementia care by people with dementia from EM groups and their informal caregivers.

### Health literacy

A total of 16 studies mentioned health literacy among people with dementia from EM groups and their informal caregivers. A lack of knowledge was reported about dementia before the diagnosis and, in general, people have not heard of dementia before their relative developed the disease (see Table 1 studies: 1, 2, 6, 8, 9, 10, 16, 17, 19, and 22). Some languages have no (easy) translation of dementia (2 and 18) and some people were unfamiliar with the words dementia or Alzheimer’s disease (14). A lack of knowledge about dementia as a health condition and memory loss was perceived as normal age-related behavior (2, 3, 5, 16, 17, 20, and 24). The first symptoms noticed by informal caregivers were memory loss/forgetfulness (1 and 5), increasingly aggressive behaviors, hallucinations (1), increasingly absent-minded, disoriented behavior and behaving strangely (17),and fluctuations in the ability to perform everyday activities (8).

### Health beliefs

One of the most common beliefs among EM groups was that symptoms of dementia are part of normal aging (1, 3, 13, 17, 19 & 24) and other physical conditions, or a reaction to a change in physical surroundings (1, 6, 15, 17, 19 and 24). A lack of knowledge about dementia within the community led to stigmatization and isolation of informal caregivers (9). Most of these studies point out that stigma results from the belief that dementia symptoms have spiritual explanations, for example that it is a punishment by God (1, 8, 14 and 24). Stigma can cause low uptake of services (21) or difficulty accepting the diagnosis (15). Others believe that dementia is a condition given by God (3 and 8) and can be cured by God (24). One example of differences in health beliefs between different EM groups is the explanations people gave for their relative’s symptoms. There are considerable differences between EM groups in ascribing the causes of dementia (1 and 24). Ideas of blaming for the symptoms on something someone did in the past were more prevalent in the South Asian group compared to the African/Caribbean group (1). Another study showed that Turkish and Moroccan informal caregivers ascribe the cause of dementia relatively often to non-physical aspects like having a difficult life whereas Surinamese Creole informal caregivers mention physical aspects like dehydration (24). One study showed that South Asian people who have less GP consultation more frequently saw the cause of dementia to be God-given, compared to South Asian people who consult GP more (7).

### Trust & expectations

A total of 13 papers mention trust in formal services among people with dementia (2, 3, 5, 6, 8, 10, 16, 17, 20, 22, and 23). The most important reason for reduced trust in formal services mentioned was an experienced lack of cultural sensitivity due to cultural differences between the person with dementia and the mainstream services (2, 3, 6, 8, 17, 20, 22, and 23). Some caregivers mentioned delays to service uptake because of a lack of trust in formal services (10 and11). People from EM backgrounds believe that the GP could not or was not willing to help (10), and even after the diagnosis family caregivers did not automatically believe the doctors (8). The expectation of persons with dementia and the families that care should be given by informal caregivers is mentioned in four papers (5,8,15, and 23) and some mention that family care is superior to formal care services (8 and 23).

## Health care needs: approachability (supply)

This scoping review includes six papers that discuss two common themes within the approachability of care. The first theme is communication, and the second theme is knowledge about resources.

### Communication

Communication with people with dementia from EM backgrounds and informal caregivers is discussed in all six papers (Table 1), especially the difficulties experienced by professionals in communication with EM families. All studies describe the language barrier experienced between the professional and the EM persons with dementia and their informal caregiver (3, 4, 6, 18, 21, and 22). One study shows that a language barrier can lead to reluctance to seek and accept help (4). Language barriers result in poor communication between professionals and persons with dementia from EM groups and their families, making it difficult to develop a good relationship with the persons’ family and to connect with the person with dementia (4). Some professionals state that there is a lack of staff who speak the languages of the EM groups (6). But one study demonstrated that even when the same language was spoken, knowledge is needed about when, with whom, and in which context to use the same language (4).

### Knowledge about resources

In two of the seven studies, professionals point out that they have insufficient knowledge and information about existing resources such as translated information material, language- and culture-sensitive diagnostic tools and knowledge of specialized dementia care and support services for EM communities (19 and 21).

## Health care seeking: ability to seek (demand)

The ability to seek dementia care is discussed in 20 studies. They show the influence of personal and social values, culture and knowledge about health care options on the access to dementia care for persons with dementia with EM backgrounds and their informal caregivers.

### Personal and social values

A total of 14 studies mention personal and social values regarding care. For some informal caregivers it is important to care for their loved ones with dementia because of cultural or religious reasons (3, 6, 12, 13, 14, 15, 17, 20, and 23). Informal caregivers had concerns about what others in the community might think if they were to break with the tradition of providing informal care (17). Keeping the caregiving task within the family was essential and provided peace of mind (13) and informal caregivers thought that outside help may be intrusive (14 and 16) and would indicate failure as the dutiful child (15). One study explains the differences within one EM group, where the generation views caring for your parents as an obligation and the younger generation interprets caring as ensuring that good care is provided. Providing care does also mean arranging proper care (23).

### Knowledge about health care options

Different studies show that EM groups have a lack of knowledge about health care options. Most of the informal caregivers were not aware of the extent of professional care available in dementia care (3, 9, 15, 16, and 22), and informal caregivers thought of mainstream support services as leading to nursing homes (14). When people from EM communities do try to get access to health care, they experience the process of getting access as difficult and feel like they are being passed from pillar to post (6). Some informal caregivers and people with dementia encounter health care accidentally, for example during hospitalization for other health issues (3).

## Health care seeking: acceptability (supply)

Four papers describe results that fit the acceptability dimension of the conceptual framework of Levesque et al. (2013). These studies show that cultural and social factors can influence the professionals’ attitude towards EM persons with dementia and their informal caregivers (3, 5, 7, and 16). The subthemes stereotypical thinking and different beliefs regarding dementia are part of the acceptability of dementia care and are described below.

### Stereotypical thinking

Three studies mention that stereotypical thinking by professionals may hinder the continuity of care (3, 4, and 6). In one study, most professionals assumed that informal care was a duty in Moroccan culture that was not be questioned (3). But informal caregivers themselves presented the originally religious and culture-inspired reasons to provide informal care blended with more pragmatic reasons (3). Professionals attributed the reluctance to seek and accept professional care to the cultural background of the EM families and professionals were talking about “they take care of their own” (4). To avoid this, professionals argued that services must respond to people as individuals (6).

### Different beliefs regarding dementia

Three studies argued that professionals are aware that not everybody interprets dementia symptoms in the same way. Behaviors considered “dementia symptoms” by professionals, may either be seen by families as normal behavior or accepted as an understandable and expected sign of old age (20). Presenting dementia as a medical condition was widely favored in one study. The professionals felt that people were more likely to understand and accept that dementia was a physical illness and that this would be less stigmatizing (6). One study showed that investing time to understand the caregivers’ health beliefs about dementia led to a greater understanding of the cultural barriers and facilitators in supporting the family (15).

## Health care utilization: ability to engage (demand)

All papers point out difficulties experienced by persons with dementia from EM groups and their informal caregivers trying to gain access to and subsequently continue to receive care from professional health care services. Some professionals report seeing EM persons in an advanced stage of dementia (18). When EM people access health care, informal caregivers experience obstacles in their relative with dementia continuing to receive formal care. Informal caregivers point out that they often felt a person-centered approach was lacking due to a lack of (cultural) sensitivity (3). Internal factors within the EM communities can also influence the decision not to continue care. The most common explanation for not using services in one study was that help from outside creates even more shame and threatens the inner pride of EM people (14). Some informal caregivers, for example, have accepted help from services like home care and nursing homes, but held on to some of the caring tasks because they wanted to show that they stayed involved (20).

## Health care utilization: appropriateness (supply)

Seven papers discuss the appropriateness of dementia care and explain difficulties in offering appropriate dementia care to persons with dementia from EM backgrounds and their informal caregivers, including lack of competencies, collaboration with the family member(s) and continuity of care (2, 3, 4, 6, 15, 20, and 21).

### Lacking competencies

In five studies, professionals indicate they do not have the competencies required to work with people with dementia from EM groups and their informal caregivers (3, 4, 6, 18, and 21). Professionals were unaware that the absence of culture- and religion-sensitive care was a primary reason for informal caregivers to avoid using professional care (3). Professionals believed that having more knowledge about the persons and their culture would help them resolve feelings of uncertainty (3) and that they need training on meeting the care needs of EM people with dementia. The professionals pointed out the importance of being better informed about dementia, recognizing behaviors associated with this disease, and needing more information about cultural and religious practices that impact persons’ behavior and daily routines (6 and 18). Professionals fear that they are not able to respond to someone’s needs because they are from a different background, although they make an effort of treating everybody with respect for individual needs and preferences. This fear and lack of knowledge may paralyze professionals (6). Even though some worked in areas with a high density of EM groups, professionals still had limited experience with persons from EM backgrounds with cognitive impairment or dementia (21). Professionals who reported having experience with EM groups also stated experiencing fewer challenges (18).

### Collaboration

Professionals find it hard to establish contact with families who often prefer to take care of their family member themselves without help from public services (18). Professionals feel insecure and hesitant because of their limited experience with EM groups in combination with a language barrier (21). One study showed the impact of ethnicity on the participants’ experiences of collaboration. Ethno-political positioning negatively impacted collaboration between colleagues and on collaboration between formal and family caregivers in home-based care (11). This study explains the common knowledge among participants: cooperation with families is more accessible when there is a shared ethnic background, even when they do not speak each other’s language (11).

### Continuity of care

After being diagnosed with dementia, continuity of dementia care is not guaranteed. In one study, professionals mention the need to improve available regular services to make them more accessible and responsive to the individual needs of persons (6). However, professionals framing self-support as a cultural custom increased the barriers to offering help (4). Uptake of services focusing on practical support seems to be least affected by EM groups, as people from various backgrounds used practical support equally. Yet, the uptake of personal care and support like home care, respite care, and nursing homes were most frequently perceived to be affected by an EM background (18). Professionals were divided on whether it was more appropriate to develop specialized services or whether the focus should be on improving available regular services to make them more accessible and able to respond to the needs of persons with an EM background (6).

## Discussion

We reviewed studies that reported on the potential barriers and facilitators in the process of access and use of health care by EM people with dementia, their informal caregivers and health care professionals. Based on the framework of Levesque et al. (2013) we found three themes related to the potential barriers on the demand and the supply sides of access to care: *health care needs, health care seeking, and health care utilization.*

First, we found a mismatch between the demand side and the supply side of access to health care. This is primarily related to a lack of abilities to perceive a need for health care of those who need care. These missing abilities are related to a lack of health literacy, beliefs about non-biological causes of dementia, and preferring informal care. When dementia is not perceived as a medical disorder, people will not perceive a need for health care. This inability to perceive a need for health care is also supported by recent studies. For example, Nielsen et al. (2020) found a lack of knowledge about dementia in EM groups posing a significant barrier for recognizing cognitive symptoms and perceiving a need for dementia care. Also, people from EM groups felt that they lacked the necessary information about dementia (Kenning et al., 2015). In line with this scoping review, other studies also found that symptoms of dementia were often attributed to a normal process of aging (Johl et al., 2016; Kenning et al., 2017) or psychosocial, physical and mental problems (Hossain et al., 2020).

The second conclusion is that persons with dementia, informal caregivers and professionals actually match with regard to their views on the search for health care. Persons with dementia from EM groups and their informal caregivers can feel a familial, cultural or religious responsibility to look after the person with dementia. This is, in fact, also the observation of many professionals who assume that caregiving tasks will be solved within the family or the community. As reported in other studies, a lack of knowledge about options in health care can cause barriers for seeking health care (Chaouni et al., 2020; Kenning et al., 2017; Nielsen et al., 2020; Parveen et al., 2017). Professionals were aware of the non-use of dementia services and assumed that these families would not consider professional care options and therefore did not suggest available professional care to them (Chaouni et al., 2020). Several professionals and informal caregivers mentioned that information about the existence of formal dementia care services rarely reached people from EM groups (Chaouni et al., 2020; Nielsen et al., 2020). However, both groups upholding these beliefs impede an active search for health care. This search is also hindered by the mismatch between demand and supply: EM groups lack knowledge about health care options, while the professionals think they are easy to find.

The third conclusion is that, once engaged in health care other barriers to the utilization of care emerge. The most often reported barriers are the lack of attention to cultural or religious backgrounds in health services and the lack of competencies among professionals to cope with differences in language and culture. This finding aligns with recent findings of Chaouni et al., (2020) and Nielsen et al. (2020), who showed that dementia care services did not fit the cultural needs of EM groups and informal caregivers felt an intercultural person-centered approach was often lacking. The study of Kenning et al. (2017) highlighted in their study that services often lacked cultural awareness and diversity for interacting with different cultural communities. The mismatch here is likely to continue to exist as professionals assume that they provide person-centered care, which reinforces their beliefs that they are in fact dealing with cultural differences and thus doing the right thing. The paradox here is that many persons from EM backgrounds feel that their needs are not being met properly and that there is a lack of person-centered care. The conceptual framework of Levesque et al. (2013) also helped us to understand the differences between and within groups. For example, ideas of blame for the cause of dementia were more prevalent in South Asian informal caregivers compared to the African/Caribbean informal caregivers (Adamson, 2001). But also within EM groups, there can be diversity. Older Turkish and Moroccan informal caregivers assume that you yourself must provide the actual care, but some younger informal caregivers of Turkish and Moroccan persons with dementia see themselves as “directing” care by arranging the professional care without having to provide it themselves (van Wezel et al., 2016). Professionals working in health care should be aware of this diversity within EM groups. The main risk of mapping the experienced barriers within EM groups is generalizing results to all EM people and stereotypical beliefs among professionals. According to the Dementia in Europe Ethics Report (Alzheimer Europe, 2018), stereotypes can be an obstacle to seeing the real person and may lead to negative stereotyping. Berdai Chaouni and De Donder (2018) showed that a lack of culture-sensitive and person-centered approach together with stereotyping and racism among professionals result in delays in the utilization of dementia care. Therefore, it is important to acknowledge the fact that there can be diversity between and within EM groups. Hossain & Khan (2018) emphasized the importance of variety in religious, ethnic, geographical, and linguistic differences. Insights and knowledge should focus on what people from EM groups have in common but not rule out individual differences between and within a particular group (Alzheimer Europe, 2018).

### Strengths and limitations

An important feature of this scoping review was the application of a conceptual framework (Levesque et al., 2013) in order to map results. In particular, comparing the care demand side and the care supply side within one dimension provides the opportunity to discover mismatches between both sides. Many papers in our scoping review did not use a theoretical model and discussed only pieces of the puzzle of care trajectories of EM persons and caregivers. Applying the framework of Levesque helped us aggregate the insights of multiple studies and organize them in the five dimensions and gain a more compete overview. We recommend the use of the framework in empirical studies to corroborate our findings from the scoping review. However, one possible limitation of using an existing framework is potentially overlooking other themes. To avoid this, all authors looked independently for (sub)themes that would also enrich the framework. No new themes were found. Another strength of this scoping review was our attention for the methodological quality of the studies. We used the MMAT (Hong et al., 2018) to add the qualitative appraisal to the process of a scoping review. A possible limitation of the use of the MMAT could be that studies with important insights were excluded. We studied the conclusions and insights of the excluded studies, but found no important or new insights.

### Implications for research and practice

Based on this scoping review, EM groups with dementia and their informal caregiver need more knowledge about the symptoms and causes of dementia to improve their ability to perceive a need for health care. This can help them recognize dementia as a disease and additionally receive support from formal and informal caregivers. The study of van Wezel et al. (2020) showed that peer group-based education enhances knowledge about dementia among EM groups. Their results also indicate that educational peer group intervention can result in more formal support. Intervention can raise awareness on options for formal support and on how to organize formal support (van Wezel et al., 2020). It is therefore important to inform and educate informal caregivers of people with dementia from EM groups to improve their ability to perceive a need for health care and their ability to seek for health care. This scoping review also showed that professionals need more education and knowledge on working with people with dementia and their informal caregivers from EM groups. A lack of knowledge and education can cause barriers for professionals in providing access to health care. Education of health care professionals can contribute to more person-centered care with respect to the cultural, religious, and linguistic needs of each person and his or her family or network.

## Conclusion

We conclude that multiple barriers were experienced in access to care for EM people with dementia and their informal caregivers. This scoping review reveals the mismatch between the demand side and the supply side of access to care. People from EM groups with dementia and their informal caregivers need better abilities to perceive, seek, and engage in healthcare. Health care professionals need to grow their insights and knowledge to strengthen their competencies to support EM groups in health care needs, health care seeking, and health care utilization. This may increase appropriate health care use by EM people with dementia and their informal caregivers.

## Appendix

**Table A1.** Search strategy.

Search	PubMed Query – October 31, 2019	Items found
#4	#1 AND #2 AND #3	790
#3	"Nursing"[Mesh] OR "nursing" [Subheading] OR "Nursing Care"[Mesh] OR "Nurses"[Mesh] OR "Home Care Services"[Mesh] OR "Primary Health Care"[Mesh] OR "Caregivers"[Mesh] OR nurs*[tiab] OR primary care[tiab] OR primary healthcare[tiab] OR primary health care[tiab] OR casemanager*[tiab] OR case manager*[tiab] OR home care[tiab] OR home healthcare[tiab] OR home health care[tiab] OR informal care[tiab] OR caregiver*[tiab] OR care giver*[tiab] OR carer*[tiab]	932547
#2	"Transients and Migrants"[Mesh] OR "Emigrants and Immigrants"[Mesh] OR "Ethnic Groups"[Mesh:NoExp] OR "Cultural Diversity"[Mesh] OR "Cultural Competency"[Mesh] OR "Culturally Competent Care"[Mesh] OR migrant*[tiab] OR immigrant*[tiab] OR emigrant*[tiab] OR minorit*[tiab] OR ethnic*[tiab] OR cultural diversit*[tiab] OR culturally competent*[tiab] OR culturally appropriate[tiab] OR cultural sensit*[tiab] OR multicultural[tiab] OR multi-cultural[tiab] OR transcultural[tiab] OR trans-cultural[tiab] OR intercultural[tiab] OR inter-cultural[tiab] OR cross-cultural[tiab] OR crosscultural[tiab] OR cultural competent*[tiab] OR turkey[tiab] OR Turkish[tiab] OR morocco[tiab] OR Moroccan*[tiab]	338927
#1	"Dementia"[Mesh] OR "Cognitive Dysfunction"[Mesh] OR alzheimer*[tiab] OR dementi*[tiab] OR mild cognitive*[tiab] OR MCI[tiab] OR mild neurocognitive[tiab] OR cognitive decline*[tiab]	267471
